# Derivation and validation of a machine learning risk score using biomarker and electronic patient data to predict progression of diabetic kidney disease

**DOI:** 10.1007/s00125-021-05444-0

**Published:** 2021-04-02

**Authors:** Lili Chan, Girish N. Nadkarni, Fergus Fleming, James R. McCullough, Patricia Connolly, Gohar Mosoyan, Fadi El Salem, Michael W. Kattan, Joseph A. Vassalotti, Barbara Murphy, Michael J. Donovan, Steven G. Coca, Scott M. Damrauer

**Affiliations:** 1 Department of Medicine, Icahn School of Medicine at Mount Sinai, New York, NY USA; 2 Renalytix AI Plc, Cardiff, UK; 3 Renalytix AI, Inc., New York, NY USA; 4 Department of Pathology, Icahn School of Medicine at Mount Sinai, New York, NY USA; 5 Department of Quantitative Health Sciences, Lerner Research Institute, Cleveland, OH USA; 6 Department of Surgery, Perelman School of Medicine at University of Pennsylvania, Philadelphia, PA USA

**Keywords:** Biomarkers, Diabetic kidney disease, Electronic data, Machine learning, Prediction

## Abstract

**Aim:**

Predicting progression in diabetic kidney disease (DKD) is critical to improving outcomes. We sought to develop/validate a machine-learned, prognostic risk score (KidneyIntelX™) combining electronic health records (EHR) and biomarkers.

**Methods:**

This is an observational cohort study of patients with prevalent DKD/banked plasma from two EHR-linked biobanks. A random forest model was trained, and performance (AUC, positive and negative predictive values [PPV/NPV], and net reclassification index [NRI]) was compared with that of a clinical model and Kidney Disease: Improving Global Outcomes (KDIGO) categories for predicting a composite outcome of eGFR decline of ≥5 ml/min per year, ≥40% sustained decline, or kidney failure within 5 years.

**Results:**

In 1146 patients, the median age was 63 years, 51% were female, the baseline eGFR was 54 ml min^−1^ [1.73 m]^−2^, the urine albumin to creatinine ratio (uACR) was 6.9 mg/mmol, follow-up was 4.3 years and 21% had the composite endpoint. On cross-validation in derivation (*n* = 686), KidneyIntelX had an AUC of 0.77 (95% CI 0.74, 0.79). In validation (*n* = 460), the AUC was 0.77 (95% CI 0.76, 0.79). By comparison, the AUC for the clinical model was 0.62 (95% CI 0.61, 0.63) in derivation and 0.61 (95% CI 0.60, 0.63) in validation. Using derivation cut-offs, KidneyIntelX stratified 46%, 37% and 17% of the validation cohort into low-, intermediate- and high-risk groups for the composite kidney endpoint, respectively. The PPV for progressive decline in kidney function in the high-risk group was 61% for KidneyIntelX vs 40% for the highest risk strata by KDIGO categorisation (*p* < 0.001). Only 10% of those scored as low risk by KidneyIntelX experienced progression (i.e., NPV of 90%). The NRI_event_ for the high-risk group was 41% (*p* < 0.05).

**Conclusions:**

KidneyIntelX improved prediction of kidney outcomes over KDIGO and clinical models in individuals with early stages of DKD.

**Graphical abstract:**

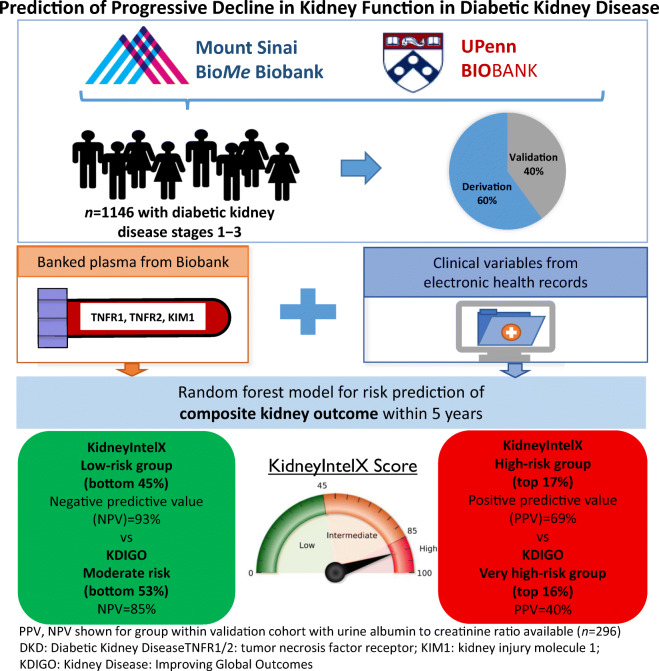

**Supplementary Information:**

The online version contains peer-reviewed but unedited supplementary material available at 10.1007/s00125-021-05444-0.

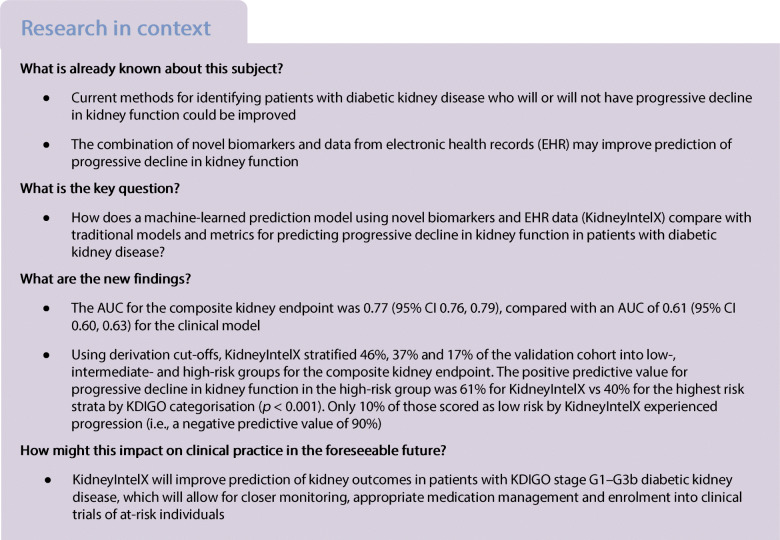



## Introduction

Approximately one out of four adults with type 2 diabetes mellitus has kidney disease (i.e., diabetic kidney disease [DKD]). Each year, 50,000 individuals with DKD progress to kidney failure in the United States [1]. The Mount Sinai Health System alone provides care for over 70,000 patients with DKD. Measurements of eGFR and urinary albumin creatinine ratio (uACR) have been incorporated into the Kidney Disease: Improving Global Outcomes (KDIGO) guidelines for risk stratification [2], but these cannot precisely identify patients who will experience rapid kidney function decline (RKFD) [3]. As a result, primary care physicians and diabetologists are not able to appropriately risk stratify and counsel patients on the progressive nature of DKD. Easily interpretable and accurate prognostic tools that integrate into clinical workflow are lacking, resulting in suboptimal treatment and delays in referral to a nephrology specialist. This has led, in part, to the unacceptable burden of progressive DKD and kidney failure [4–8] with a high proportion of patients starting unplanned dialysis [1, 9, 10].

Several blood-based biomarkers have shown associations with DKD progression, most significantly soluble TNF receptors 1/2 (TNFR1/2) and plasma kidney injury molecule-1 (KIM-1) [11, 12]. However, accurate prognostic models that combine clinical data from patients’ electronic health records (EHR) with blood-based biomarkers have not been implemented. Although EHR data are widely available, its volume and complexity limits integration with biomarker values using traditional methodologies. Machine learning can combine biomarkers and EHR data to produce prognostic risk scores. We previously demonstrated that combining biomarkers and EHR data in patients with type 2 diabetes and *APOL-1* high-risk genotype improved prediction of kidney outcomes over clinical models [13]. A simple risk score that improves the ability to identify patients with DKD at low, intermediate, and high risk of progressive decline in kidney function has the potential to improve outcomes through more effective use of medications and efficient resource allocation at the primary care physician level.

In this study, we sought to develop and validate the performance of a new biomarker-enriched, machine-learned risk score (the KidneyIntelX™ test) to predict progressive decline in kidney function in patients with early-stage DKD and compare the performance with standard clinical models. We also determined risk-based thresholds that can easily be integrated into standard clinical workflows and enhance existing clinical practice guidelines.

## Methods

### Study sample

**Fig. 1:**
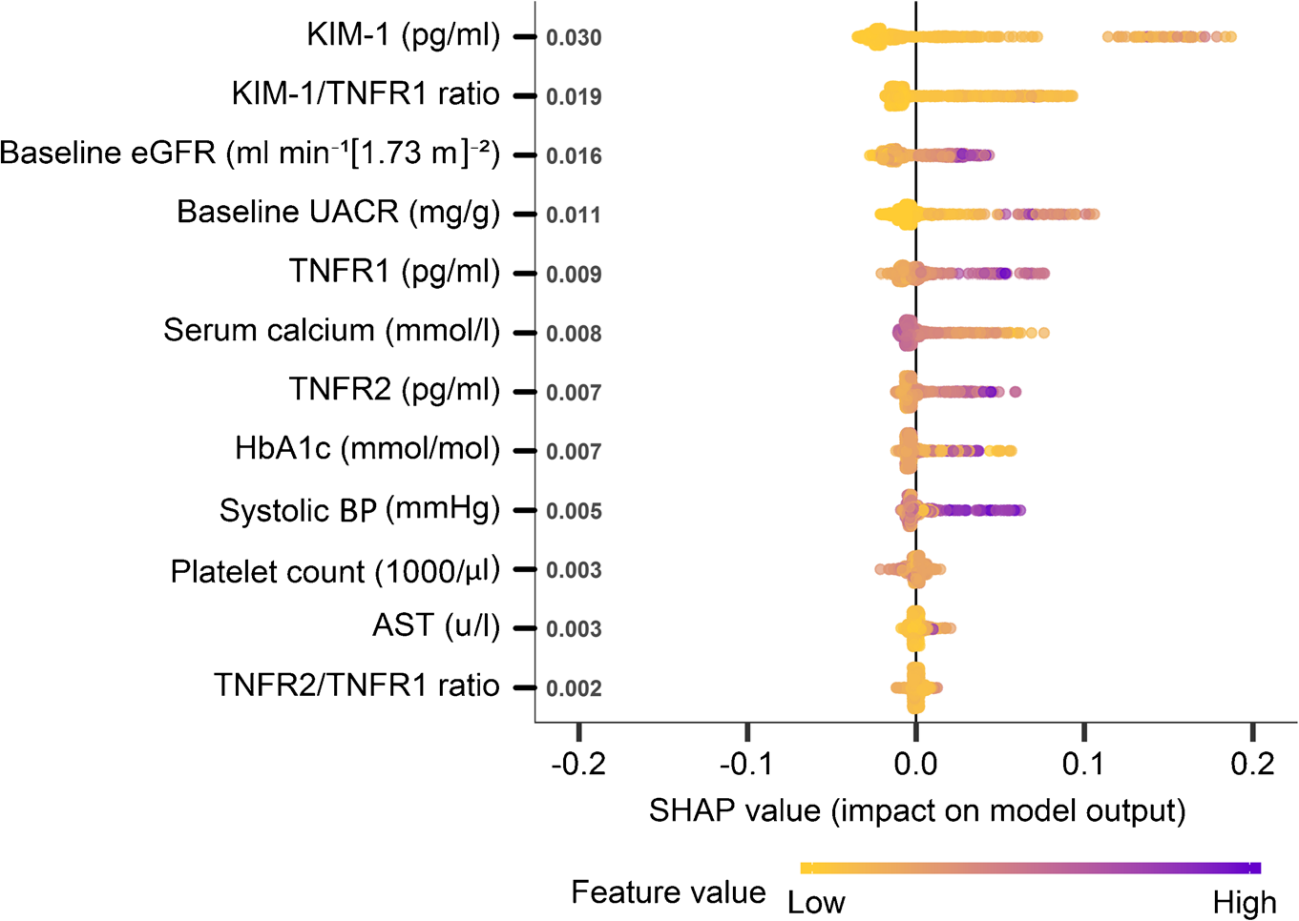
Shapley additive explanations (SHAP) plot showing relative feature importance. SHAP summary plots order features based on their importance. Each plot is made up of individual points from the training dataset with a higher value being darker purple and a lower value being more yellow. If the dots on one side of the middle line are more purple or yellow, this suggests that the values are increasing or decreasing, respectively, moving the prediction in that direction. For example, higher systolic BP is associated with higher risk of the composite kidney outcome. AST, aspartate aminotransferase

Samples were from the Bio*M*e Biobank at the Icahn School of Medicine at Mount Sinai and the Penn Medicine Biobank (PMBB). The Bio*Me* Biobank is a biorepository of plasma and DNA collected from 2007 from individuals in a diverse local community in New York City. Informed consent for access to the patients’ EHR is also included [14, 15]. PMBB is a biobank of blood and tissue samples taken from a research cohort recruited via the University of Pennsylvania Health System from 2008 [14]. Participants gave consent for their biospecimens to be linked with their longitudinal EHR (electronic supplementary material [ESM] Fig. 1). Both Bio*Me* and PMBB are institutional biobanks that attempt to be representative of the patient populations of the institutions they serve. Patients are recruited from outpatient general medicine clinics and certain subspecialty clinics with limited pre-selection criteria [16, 17].

The study protocol was approved by each institution’s review board; all participants had provided written informed consent to participate in research and were not specifically compensated for participation in the current study. Blood was collected on the day of enrolment into Bio*Me* or PMBB and plasma was isolated as per standard procedures and continuously stored at −80°C until shipping to the RenalytixAI laboratory, USA where biomarkers were measured.

### Inclusion criteria

We selected patients from Bio*Me* and PMBB who were 21–81 years at the time of biobank enrolment (‘baseline’), with type 2 diabetes, an eGFR between 30 and 59.9 ml min^−1^ [1.73 m]^2^ or an eGFR ≥60 ml min^−1^ [1.73 m]^2^ with uACR ≥3 mg/mmol. The KDIGO risk model categorises patients based on eGFR and albuminuria and has three colours that correspond to the prognosis of prevalent CKD (we did not include patients at ‘low risk’ or green because they do not have CKD) [2]. Patients were included if, by the KDIGO eGFR and uACR criteria, they were stage G3a–G3b with all grades of albuminuria (A1–A3) and stage G1–G2 with moderate to high albuminuria (uACR ≥30 mg/g [A2–A3]) [2]. The proportion of each DKD stage was evaluated against national estimates derived from the National Health and Nutrition Examination Survey (NHANES) 2018–2019 [18]. For eGFR, we defined the baseline period as 1 year before or up to 3 months after biobank enrolment. Baseline uACR values were derived from closest values ±1 year from enrolment to maximise sample size as these are measured less frequently; participants without baseline values of eGFR and uACR meeting these criteria were excluded. Only individuals with a stored plasma specimen, a minimum follow-up time from enrolment of at least 21 months, at least three eGFR values after baseline (ESM Fig. 1) were included. Individuals with kidney transplants or on chronic maintenance dialysis before baseline were excluded from the study.

### Ascertainment of clinical variables

Data on sex and race were obtained from the Bio*Me* and PMBB biobanks or from EHR data. Clinical data were extracted for all EHR variables with concordant time stamps. Hypertension and type 2 diabetes status at baseline were determined using the eMERGE Network phenotyping algorithms [16]. CVD and heart failure were determined by ICD-9/10 clinical modification codes.

### Biomarker assays

The three plasma biomarkers were measured in a proprietary, analytically validated multiplex format using the Mesoscale platform (MesoScale Diagnostics, Gaithersburg, Maryland, USA), which employs electrochemiluminescence detection methods combined with patterned arrays to multiplex assays. Each sample was run in duplicate, along with quality control samples with known low, moderate and high concentrations of each biomarker on each plate. Assay precision was assessed using a reference panel of seven samples that spanned the measurement range. Intra-assays for KIM-1, TNFR1 and TNFR2 gave mean CV values of 3.9%, 5.4%, and 3.7%, respectively. Inter-assays for KIM-1, TNFR-I, and TNFR-2 reference samples gave mean CV values of 9.9%, 10.1%, and 7.8%, respectively. Assays satisfied dilution linearity and were run at 1:4 dilution. Levey–Jennings plots were employed and followed the Westgard rules for re-run of samples. The laboratory personnel performing the biomarker assays were blinded to all clinical information.

### Data harmonisation

We harmonised data from Bio*Me* and PMBB biobanks. Race/ethnicity was collapsed into four major, non-overlapping categories (White, Non-Hispanic Black, Hispanic, and other). ICD and Current Procedural Terminology (CPT) codes were included as yes/no variables with timestamps. Medications (including metformin, insulin, sulfonylureas, etc. that were prescribed before the baseline data) were mapped to RxNorm codes [19] and laboratory values to Logical Observation Identifiers Names and Codes (LOINC) codes [20]. Only variables represented in >70% of participants throughout the combined dataset (except uACR and BP because of their established clinical importance) were included and used to train the KidneyIntelX algorithm.

### Ascertainment and definition of the kidney endpoint

We determined eGFR using the CKD-EPI creatinine equation [21]. We employed linear mixed models with an unstructured variance-covariance matrix and random intercept/slope for each individual to estimate the eGFR slope [22]. The primary composite outcome, progressive decline in kidney function, included the following: RKFD defined as an eGFR slope decline of ≥5 ml min^−1^ [1.73 m]^−2^ per year [2], a sustained (confirmed at least 3 months later) decline in eGFR of ≥40% [23] from baseline, or ‘kidney failure’ defined by sustained eGFR <15 ml min^−1^ [1.73 m]^−2^ confirmed at least 30 days later, or receipt of long-term maintenance dialysis or receipt of a kidney transplant [2]. Additionally, two nephrologists (SC, GNN) independently adjudicated all outcomes examining each individual over their longitudinal course, accounting for eGFR changes (ensuring annualised decline of ≥5 ml/min or ≥40% sustained decrease), corresponding ICD/CPT codes and medications to ensure that outcomes represented true decline rather than a context dependent temporary change (e.g., due to medications/hospitalisations). Follow-up time was censored after loss to follow-up, after the date that the non-slope components of the composite kidney endpoint were met, or 5 years after baseline.

### Statistical analysis

The datasets were randomised into derivation (60%) and validation sets (40%). The validation dataset was completely blinded and sequestered from the total derivation dataset. Using only the derivation set, we evaluated supervised random forest algorithms on the combined biomarker and all structured EHR features without a priori feature selection and identified a candidate feature set using grid search; ESM Table 1. The derivation set was then randomly split into secondary training and test sets for model optimisation with 70%–30% spitting and a tenfold cross-validation for AUC. We considered both raw values and ratios of the biomarkers. Missing uACR values were imputed to 1.1 mg/mmol [24], missing BP values were imputed using multiple predictors (age, sex, race and antihypertensive medications) [25], and median values were used for other features where missingness was <30% (ESM Table 2).

We conducted further iterations of the model by tuning the individual hyperparameters. A hyperparameter is a parameter that is used to control the learning process (e.g., number of random forest trees) as opposed to parameters whose weights are learned during the training (e.g., weight of a variable). Tuning hyperparameters refers to iteration of model architecture after setting parameter weights to achieve the ideal performance. Hyperparameters were optimised using the grid search approach. K-fold cross-validation-based AUC was evaluated for all possible combinations of hyperparameters. We selected the combination of hyperparameters that optimised the AUC for model building. The following hyperparameters were considered for optimisation.

- Number of variables randomly selected as candidates for splitting a node
- Mean forest number of unique cases (data points) in a terminal node
- Maximum depth to which a tree should be grown

The code for hyperparameter optimisation has been deposited in a github repository (https://github.com/girish-nadkarni/KidneyIntelX_hyperparameter_tuning) to improve reproducibility and transparency. The final model was selected based on AUC performance.

We generated risk probabilities for the composite kidney endpoint using the final model in the derivation set, scaled them to align with a continuous score from 5 to 100 by increments of 5, and applied this score to the validation set. Risk cut-offs were chosen in the derivation set to encompass the top 15% as the high-risk (scores 90–100), bottom 45% as the low-risk (scores 5–45), and the intervening 40% as the intermediate-risk group (scores 50–85). Primary performance criteria were AUC, positive predictive value (PPV) for high-risk group and negative predictive value (NPV) for low-risk group at the pre-determined cut-offs. The selected model and associated cut-offs were then validated by an independent biostatistician (MK) in the sequestered validation cohort.

In addition to these traditional test statistics, we assessed calibration by examining the slope of observed vs expected outcome plots of the KidneyIntelX score vs only the observed outcomes. We also constructed Kaplan–Meier curves for time-dependent outcomes of 40% decline and kidney failure with HRs using the Cox proportional hazards method.

The discrimination of the KidneyIntelX model was compared with a recently validated comprehensive clinical model that included age, sex, race, eGFR, CVD, smoking, hypertension, BMI, uACR, insulin, diabetes medications, and HbA_1c_ and was developed to predict 40% eGFR decline in individuals with type 2 diabetes [24]. Utility metrics (PPV, NPV) were compared with both the comprehensive clinical model and KDIGO risk strata. We also calculated the net reclassification index (NRI) for events and non-events compared with KDIGO risk strata [26, 27]. Finally, we compared the validated KidneyIntelX model with a logistic regression model incorporating the features found to be significantly driving the outcome. All a priori levels of significance were <0.05. All hypothesis tests were two-sided. 95% confidence intervals were calculated by bootstrapping. All analyses were performed with R software (www.rproject.org), the dplyr package, the randomForestSRC and the CARET package [28, 29].

## Results

### Baseline characteristics of cohorts

Baseline characteristics of the total study cohort (*n* = 1146) were as follows: median age 63 years, 581 (51%) female, median eGFR 54 ml min^−1^ [1.73 m]^−2^, and median uACR 6.9 mg/mmol. uACR was available in 62% of the cohort and imputed to 1.1 mg/mmol in 38%. The most common comorbidities were hypertension (91%), CAD (35%), and heart failure (33%). The majority (81%) were on ACE inhibitors or angiotensin receptor blockers. Baseline characteristics between derivation and validation sets including event rates were balanced. The median number of serum creatinine/eGFR values per participant during the follow-up period was 16 (Table 1). The distribution of DKD stages of the study cohort is similar to national estimates based on NHANES (ESM Table 3).

**Table 1:** Clinical characteristics of the participants in the derivation and validation cohorts

	Study population *n* = 1146	Derivation population *n* = 686	Validation population *n* = 460
Clinical characteristics			
Age in years, median [Q1–Q3]	63 [55–69]	63 [55–68]	63 [56–69]
Female, *n* (%)	581 (50.7)	352 (51.3)	229 (49.8)
Race, *n* (%)			
White	373 (32.6)	231 (33.7)	142 (30.9)
African American	386 (33.7)	226 (32.9)	160 (34.8)
Other	387 (33.8)	229 (33.4)	158 (34)
BMI, median [Q1–Q3]	31 [29–35]	31 [29–35]	31 [29–36]
Hypertension, *n* (%)	1043 (91.0)	622 (90.7)	421 (91.5)
CAD, *n* (%)	406 (35.4)	234 (34.1)	172 (37.4)
Heart failure, *n* (%)	378 (33)	213 (31.1)	165 (35.9)
Systolic BP (mmHg)	130 [120–144]	130 [119–144]	130 [120–144]
Diastolic BP (mmHg)	74 [67–81]	74 [66–81]	73 [67–80]
Follow-up (months), median [Q1–Q3]	51.9 [36.5–58.1]	51.3 [36.8–58.1]	52.8 [35.9–58.1]
Laboratory characteristics			
eGFR (ml min^−1^ [1.73 m]^−2^)			
Baseline, median [Q1–Q3]	54.3 [45.3–67.3]	54.4 [44.4–68.4]	54.1 [45.7–66.1]
30–44.9, *n* (%)	279 (24.4)	176 (25.7)	103 (22.4)
45–59.9, *n* (%)	490 (42.8)	275 (40.1)	215 (46.7)
60–89.9, *n* (%)	263 (22.9)	170 (24.8)	93 (20.2)
≥ 90, *n* (%)	114 (9.9)	65 (9.5)	49 (10.7)
uACR (mg/mmol)			
Baseline, median [Q1–Q3]	6.9 [1.8–27.2]	7.4 [2–26.9]	6.1 [1.7–27.8]
Missing, *n* (%)	433 (37.8)	269 (39.2)	164 (35.7)
Baseline HbA_1c_, median [Q1–Q3]			
mmol/mol	51.9 [44.3–66.1]	53 [44.3–66.3]	51.9 [44.3–66.1]
%	6.9 [6.2–8.2]	7 [6.2–8.22]	6.9 [6.2–8.2]
Medication			
ACEi/ARB, *n* (%)	926 (80.8)	560 (81.6)	366 (79.6)
Plasma biomarkers (pg/ml), median [Q1–Q3]			
TNFR1	2807 [2192–3830]	2807 [2191–3830]	2924 [2217–3894]
TNFR2	11,090 [8031–14,984]	11,090 [8031–14,984]	11,171 [8302–15,046]
KIM-1	124 [76–235]	124 [76–235]	138 [82–253]
Smoking status, *n* (%)			
Never	354 (30.9)	214 (31.2)	140 (30.4)
Ever	503 (43.9)	298 (43.4)	205 (44.6)
Missing	289 (25.2)	174 (25.4)	115 (25)
Events			
eGFR slope ≥ 5 ml min^−1^ [1.73 m]^−2^ per year, *n* (%)	171 (14.9)	98 (14.3)	73 (15.9)
Sustained 40% decline in eGFR, *n* (%)^a^	179 (15.6)	103 (15)	76 (16.5)
Kidney failure, *n* (%)^b^	52 (4.5)	29 (4.2)	23 (5)
Composite endpoint, *n* (%)^c^	241 (21)	137 (20)	104 (22.6)

^a^Confirmed at least 3 months later. Defined as a decline in eGFR of ≥40% from baseline

^b^Defined by sustained eGFR <15 confirmed at least 30 days later, or receipt of long-term maintenance dialysis or receipt of a kidney transplant

^c^Defined as progressive decline in kidney function defined by any of the following: eGFR slope ≥ 5 ml min^−1^ [1.73 m]^−2^ per year or sustained 40% decline in eGFR or kidney failure

ACEi, ACE inhibitor; ARB, angiotensin receptor blocker; CAD, coronary artery disease

### Prediction of the composite kidney endpoint (progressive decline in kidney function)

**Fig. 2:**
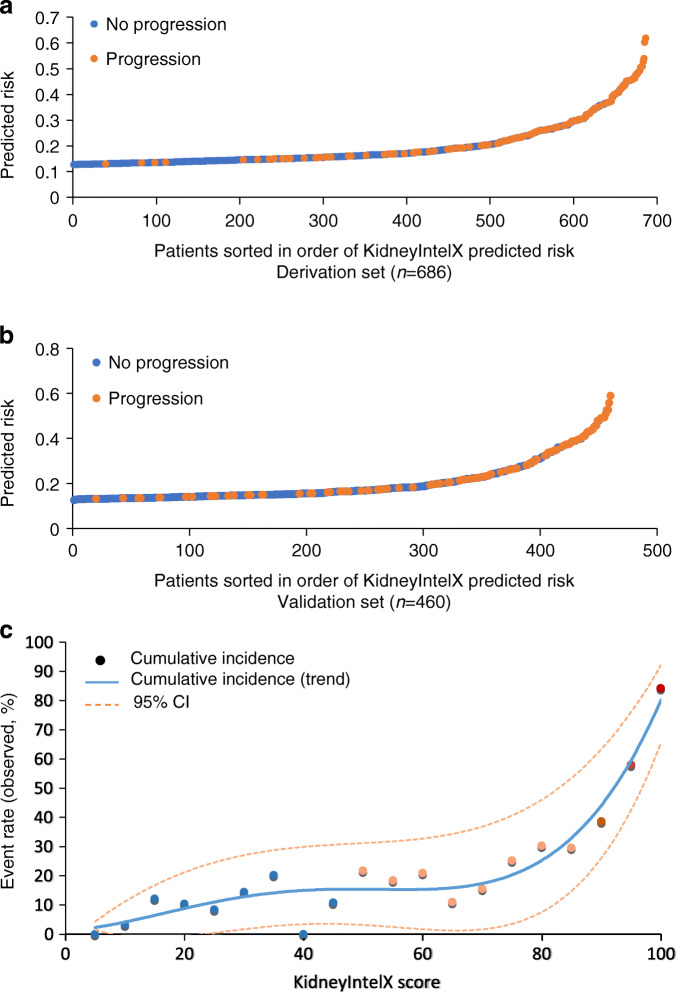
Composite kidney endpoint event rates by (**a**) KidneyIntelX predicted risk in derivation set, (**b**) KidneyIntelX predicted risk in validation set and (**c**) KidneyIntelX score prediction distributions of patients with DKD according to the risk of composite kidney endpoint in the derivation and validation set. (**a**, **b**) Events are denoted with an orange dot (progression) and represent the composite kidney endpoint within 5 years. Non-events are denoted with blue dots (no progression) and represent an absence of the composite kidney event in the follow-up period. (**c**) Dots represent cumulative incidence: blue, low risk 10% (6%, 14%); pink, intermediate risk 22% (16%, 28%); and red, high risk 61% (50%, 71%)

Overall, 241 patients (21%) experienced progressive decline in kidney function over a median 4.3 (IQR 3.0–4.8) years. In the complete derivation set (*n* = 686), using tenfold cross-validation for discrimination, the mean AUC for the KidneyIntelX model was 0.77 (95% CI 0.74, 0.79). The most significant data features contributing to performance of the KidneyIntelX model included the three plasma biomarkers (TNFR1, TNFR2 and KIM1, as discrete values and ratios), eGFR, uACR, and systolic BP (Fig. 1). This final model had an AUC of 0.77 (95% CI 0.76, 0.79) in the validation set (*n* = 460). The risk for the composite kidney event increased by predicted probabilities of the KidneyIntelX score (Fig. 2a and b) and by the KidneyIntelX score (Fig. 2c). The slope of the observed vs the predicted risk for KidneyIntelX was 0.8 in the training set and 1.0 in the validation set, indicating good calibration (ESM Fig. 2). By comparison, the comprehensive clinical model yielded an AUC of 0.62 (95% CI 0.61, 0.63) in the full derivation set (*n* = 686) and 0.61 (95% CI 0.60, 0.63) in validation set (*n* = 460; Delong *p* value for KidneyIntelX vs clinical model <0.001).

### KidneyIntelX clinical utility cut-off points

The risk probability cut-offs of KidneyIntelX selected in the derivation set (*n* = 686) were 0.061 for the lowest 45% of patients and 0.302 for the top 15% of patients. When these risk cut-offs were applied to the complete validation set, with imputed uACR for missing values (*n* = 460), KidneyIntelX stratified patients to low- (46%), intermediate- (37%) and high-risk (17%) groups with respective probabilities for the composite kidney endpoint of 0.10, 0.22 and 0.61. When the optimised clinical model was applied to the validation set, the respective probabilities for the composite kidney endpoint were 0.171 for the bottom 46% of the population and 0.319 for the top 17%. Thus, the PPV for the composite kidney endpoint was 61% in the KidneyIntelX high-risk group compared with a PPV of 37% for the comprehensive clinical model (*p* < 0.001; Table 2). The NPV for the composite kidney endpoint in the KidneyIntelX low-risk group was 90% compared with an NPV of 88% for the comprehensive clinical model (*p* = 0.33). The distribution of patients into KDIGO risk categories was established using 296 participants (64%) with uACR available in the validation cohort and stratified the population into ‘moderately increased risk’ (53%), ‘high risk’ (31%) and ‘very high risk’ (16%) with respective probabilities of 0.15, 0.29 and 0.40 for the composite kidney endpoint over 5 years. In the subgroup with non-imputed uACR (*n* = 296), the PPV for the high-risk strata of KidneyIntelX was 69% (compared with 40% for KDIGO ‘very high’ risk) and the NPV for the low-risk strata of KidneyIntelX was 93% (compared with 85% for KDIGO ‘moderately increased’ risk; ESM Table 4).

**Table 2:** Test characteristics for KidneyIntelX and the comprehensive clinical model

Predicted risk	KidneyIntelX risk score	Full derivation set (*n* = 686)^a^				Validation set (*n* = 460)^b^				Predicted risk	Optimised clinical model			
		Population	Sens	Spec	NPV/PPV	Population	Sens	Spec	NPV/PPV		Population	Sens	Spec	NPV/PPV
Low risk										Low risk				
0.040	≤30	Lowest 30%	96%	37%	98%	Lowest 32%	88%	38%	91%	0.142	Lowest 32%	74%	33%	86%
0.061	≤45	Lowest 45%	88%	53%	95%	Lowest 46%	81%	54%	90%	0.171	Lowest 46%	67%	48%	88%
0.0712	≤50	Lowest 50%	85%	59%	94%	Lowest 48%	77%	58%	90%	0.175	Lowest 48%	67%	51%	89%
High risk														
0.241	≥85	Top 20%	56%	89%	56%	Top 21%	50%	88%	55%	0.288	Top 21%	41%	82%	31%
0.302	≥90	Top 15%	46%	93%	63%	Top 17%	45%	93%	61%	0.319	Top 17%	37%	88%	37%
0.401	≥95	Top 10%	32%	96%	67%	Top 12%	31%	96%	70%	0.361	Top 12%	28%	91%	38%

^a^AUCs in derivation set: 0.85 (95% CI 0.84, 0.86) in train and AUC 0.77 (95% CI 0.74, 0.79) from tenfold cross-validation testing

^b^AUC in validation set 0.77 (95% CI 0.76, 0.79)

NPV, negative predictive value (for low risk); PPV, positive predictive value (for high risk); Sens, sensitivity; Spec, specificity

KidneyIntelX scores correctly classified more cases into the appropriate risk strata (NRI_event_ = 55% in the derivation set and 41% in the validation set, *p* < 0.05; ESM Table 5) than the KDIGO risk strata did. NRI_non-event_ was −8.2% in the derivation set and − 7.9% in the validation set (*p* = NS).

### Time-to-event analyses for 40% sustained decline or kidney failure

**Fig. 3:**
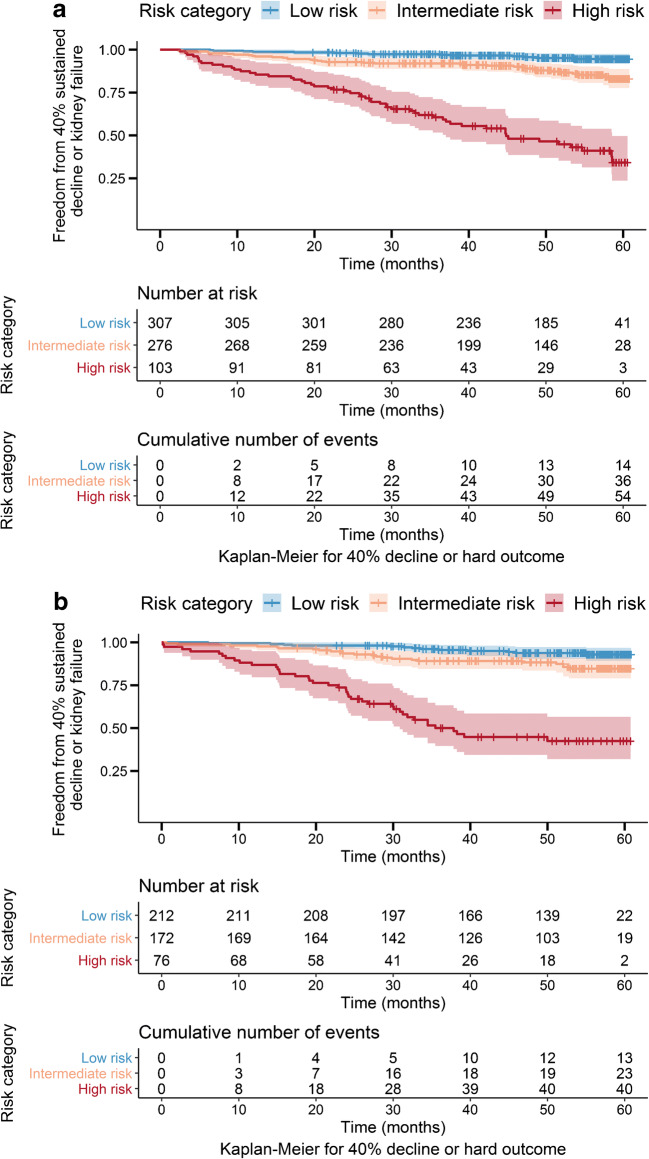
Kaplan–Meier curves by KidneyIntelX risk strata for the endpoint of sustained 40% decline in eGFR or kidney failure in derivation (**a**) and validation (**b**) sets. The risk cut-offs derived from derivation and applied to validation were: low risk 0–0.061129, intermediate risk 0.061129–0.30209 and high risk 0.30209–1. In the derivation set, 45% were low risk, 40% were intermediate risk and 15% were high risk. In the validation set, 46% were low risk, 37% were intermediate risk, and 17% were high risk. The HR for high vs low risk was 18.3 (95% CI 10.1, 33.1) in derivation and 14.7 (95% CI 7.8, 27.6) in validation. The HR for high vs intermediate risk was HR 5.7 (95% CI 3.7, 8.7) in derivation and 6.0 (95% CI 3.5, 10.0) in validation. The HR for high vs low and intermediate risk combined was 9.2 (95% CI 6.2, 13.6) in derivation and 9.1 (95% CI 5.8, 14.4) in validation

Patients with high-risk KidneyIntelX scores (top 15% in the derivation set and top 17% in the validation set) had greater risk of progression to time-to-event categorical outcomes of 40% sustained decline or kidney failure than patients in the low- or medium-risk strata combined did (HR 9.2; 95% CI 6.2, 13.6 in derivation and 9.1, 95% CI 5.8, 14.4 in the validation set; Fig. 3a and b). Kaplan–Meier curves by KDIGO risk categories in the training and validation set are shown in ESM Fig. 3.

#### Subgroup analysis

KidneyIntelX performed similarly across patients with an eGFR greater or less than 60 ml min^−1^ [1.73 m]^−2^ at baseline (0.78 and 0.76, respectively). Additionally, when only data in the year prior to enrolment were included, the AUC was identical (0.77) as was the PPV for the top 17% (61%) and the NPV for the bottom 45% (91%). Kaplan–Meier plots did not change when limited to patients with data ≥5 years to ensure that patients were alive for at least 5 years (ESM Fig. 4).

#### Comparison with logistic regression model

We compared the performance of a logistic regression model that incorporated the top 12 final features that were trained and validated in the KidneyIntelX random forest model. The AUC for a logistic model with those 12 features was 0.75, and the PPV for the top 17% of the population was 59%.

#### Discrimination for ‘kidney failure’ endpoint

Using the same KidneyIntelX model specifically trained for the composite kidney endpoint, the AUC of KidneyIntelX risk scores for the ‘kidney failure’ endpoint alone was 0.87 (95% CI 0.84, 0.89) in the derivation cohort and 0.89 (95% CI 0.87, 0.91) in the validation cohort.

## Discussion

Utilising plasma samples of individuals with type 2 diabetes from two biobanks and linked EHR data, we developed and validated a risk score combining clinical data and three plasma biomarkers via a random forest algorithm to predict a composite kidney outcome, progressive decline in kidney function, consisting of RKFD, sustained 40% decline in eGFR, and kidney failure over 5 years. We demonstrated that the KidneyIntelX outperformed models that use standard clinical variables alone, including the KDIGO risk categories [2]. There were marked improvements in discrimination over clinical models, as measured by AUC, NRI and improvements in PPV compared with KDIGO risk categories. Furthermore, we showed that KidneyIntelX accurately identified over 40% more patients experiencing events than the KDIGO risk strata did. Finally, KidneyIntelX provided good risk stratification for the accepted US Food and Drug Administration endpoint of sustained 40% decline in eGFR or kidney failure with a 15-fold difference in risk between the high-risk and low-risk strata for this clinical and objective endpoint.

DKD is an increasingly complex and common problem challenging modern healthcare systems. In real world practice, predicting DKD progression is challenging, particularly in early disease, so improving prognostic tests is paramount. Our integrated risk score has near-term clinical implications, especially when linked to clinical decision support and embedded care pathways. The current standard for clinical risk stratification (KDIGO risk strata) [2] has three risk strata that overlap with the population of DKD patients that we included in our study. We also created a risk score with three risk strata (low, intermediate and high) incorporating KDIGO classification components (eGFR and uACR), as well as other clinical variables and three blood-based biomarkers. In this way, we were able to augment the ability to accurately risk stratify patients with DKD, thereby enabling improved patient management.

Low-risk patients with DKD can continue care with their existing providers and require less intense treatments, unless repeat testing, changes in clinical status or local arrangements regarding referral to specialist care indicate otherwise. For those with high-risk scores, oversight may include more referrals to nephrology [30, 31], increased monitoring intervals, improved awareness of kidney health, referral to dieticians, reinforcement of usage of antagonists of the renin angiotensin aldosterone system, and increased motivation to start recently approved medications, including SGLT2 inhibitors and GLP-1 receptor agonists to slow progression [32, 33]. Earlier engagement with nephrologists may also allow for more time to advise and educate patients about home-based dialysis and pre-emptive or early kidney transplant as patient-centred kidney replacement options when appropriate. The use of a risk score as part of the enrolment process in future RCTs may enrich the trial participants for greater likelihood of events and thus reduce the chances for type 2 error or minimise the sample size needed to detect a statistically significant difference between treatment and control. Interventions that prevent or slow DKD progression and foster patient-centred kidney replacement modalities support the goals of the US Department of Health and Human Services’ Advancing American Kidney Health initiative [34].

KidneyIntelX included inputs from biomarkers examined in several settings. These biomarkers have demonstrated reliable independent prognostic signals for kidney function decline and ESRD [11, 12, 15, 35–38]. In our previous study, we found that including biomarkers to clinical data derived from EHR at a single-centre had better predictive performance than clinical models alone [13]. However, that study included few patients with prevalent CKD (approximately one third had CKD in the cohort with type 2 diabetes and one quarter had CKD in the APOL1 high-risk cohort). In our current study, we expanded the cohort to a second medical centre (University of Pennsylvania), and trained and validated a new model focused exclusively on patients with prevalent DKD at baseline. By incorporating biomarker concentrations and EHR data into our machine learning algorithm, we were able to provide a multidimensional representation of risk for individuals with DKD and allow for the model to generate improved prognostic estimates for future progression [39, 40]. Other biomarkers (e.g., SUPAR) and composite tests that incorporate other plasma biomarkers (apolipoprotein A-IV, CD5 antigen-like, IGF-binding protein 3) and some clinical data features have been shown to accurately predict incident CKD in individuals with type 2 diabetes; however, this does not exclude other approaches that include additional biomarkers and novel methods of data analysis [41–43]. The goal of the KidneyIntelX test is to determine which patients with established DKD are at highest risk of progressive decline in kidney function or kidney failure and those with CKD that is unlikely to progress over time.

Our study has limitations. uACR was missing in 38% of the cohort, but this is representative of current state of care [1, 44]. Moreover, our goal was to develop a risk score using real world data from EHR to predict where uACR is missing in a significant number of patients. More widespread availability of uACR values would enhance the performance of KidneyIntelX, as it was a contributing feature in our model. However, even with this limitation, KidneyIntelX had a more robust performance than the KDIGO very high-risk stratum in the subpopulation with uACR measurements. Second, there was no protocolised follow-up resulting in missing data and lack of kidney biopsies. Missing data can lead to biased machine learning models and the data are prone to ascertainment bias [45]. However, the median number of eGFR values per participant was 16, and the median time of follow-up was 4.3 years. Although the primary biobanked cohorts used in the study were broadly representative of individuals with DKD in type 2 diabetes in terms of race/ethnicity and gender, we cannot rule out an inherent bias since the recruitment was opt-in recruitment from outpatient clinics and individuals who chose to participate in the cohorts from which the study population was selected may be different from those who did not participate in the primary cohorts. Additionally, we did not have information on the participants’ socioeconomic status or the duration of the diabetes diagnosis. In the absence of biopsy, we could not exclude the possibility that CKD may be due to other causes. The test performance of KidneyIntelX (random forest algorithm) was higher than a logistic regression model that utilised the final top biomarker and clinical features that were selected by the random forest approach. However, we chose to employ the machine learning approach because random forests can integrate feature selection and modelling as well as efficiently model potential non-linear interactions between features. Finally, both cohorts are from Northeast USA and an independent validation cohort is needed to ensure generalisability. However, only one third of the participants were white, so there was adequate representation of racial groups that experience disparities for kidney disease.

In conclusion, a machine-learned model combining plasma biomarkers and EHR data significantly improved prediction of progressive decline in kidney function over comprehensive clinical models without biomarkers in individuals with DKD in type 2 diabetes from two large academic medical centres.

## Supplementary Information

ESM (PDF 1025 kb)

## Data Availability

The datasets generated during and/or analysed during the current study are available from the corresponding author on reasonable request.

## Abbreviations

CAD: Coronary artery disease
CPT: Current procedural terminology
DKD: Diabetic kidney disease
EHR: Electronic health records
KDIGO: Kidney Disease: Improving Global Outcomes
KIM-1: Kidney injury molecule-1
NHANES: National Health and Nutrition Examination Survey
NPV: Negative predictive values
NRI: Net reclassification index
PMBB: Penn Medicine Biobank
PPV: Positive predictive values
RKFD: Rapid kidney function decline
TNFR1/2: TNF receptors 1/2
uACR: Urinary albumin creatinine ratio
