# Supplementary material for: Derivation and validation of a machine learning risk score using biomarker and electronic patient data to predict progression of diabetic kidney disease
Source: Diabetologia. 2021 Apr 2;64(7):1504–15. doi: 10.1007/s00125-021-05444-0 (PMC8187208; doi:10.1007/s00125-021-05444-0)
Supplement: Supplementary file 1 — (PDF 1025 kb) [file 125_2021_5444_MOESM1_ESM.pdf]

Supplemental File

Title: Derivation and validation of a machine learning risk score using biomarker and electronic patient data to predict rapid progression of diabetic kidney disease

Tables:

1. **ESM Table 1. Candidate Feature Set**
2. **ESM Table 2. Proportion of missing values for a selection of features considered in the model.**
3. **ESM Table 3. Percent of patients in each strata of KDIGO risk from A) NHANES and B) KidneyIntelX study cohort**
4. **ESM Table 4. Test characteristics by KDIGO and KidneyIntelX Risk Categories**
5. **ESM Table 5. Net Reclassification from KDIGO Risk Strata to KidneyIntelX Risk Strata in Derivation and Validation**

Figures:

1. **ESM Fig. 1. Selection of Cohorts**
2. **ESM Fig. 2. Observed vs. Expected (calibration plot) for the Composite Kidney Outcome in Derivation (Panel A) and Validation (Panel B)**
3. **ESM Fig. 3. Kaplan-Meier Curves by KDIGO Risk Strata for the Endpoint of Sustained 40% Decline in eGFR or Kidney Failure in Derivation (Panel A) and Validation (Panel B)**
4. **ESM Fig. 4. Kaplan-Meier Curves by KidneyIntelX Risk Strata for the Endpoint of Sustained 40% Decline in eGFR or Kidney Failure in Validation (Panel A) and in patients with data ≥ 5 years (Panel B)**

ESM Table 1. Candidate Feature Set

Feature Category	Number of Features	Description
**Baseline Demographics**	5	Age, Sex, Race
**Laboratory Values**	27<br>3 Plasma Biomarkers	All lab values (linked to LOINC codes) that were present in a minimum of 70% of subjects in the study<br>Biomarkers measured on all, ratios of plasma biomarkers
**ICD 9 / 10**	20	Top 20 represented codes from the subject population
**Medications**	30	Top 30 medications (linked to RxNorm) represented in the study population
**Vital Signs**	3	Systolic Blood Pressure, Diastolic Blood Pressure, BMI
**Total**	>100 based on derived features	Derived features on lab values including min, max, median, slope, ratio of Biomarkers

ESM Table 2. Proportion of missing values for a selection of features considered in the model.

Candidate Feature	Missing proportion
Age	0%
Sex	0%
Race	0%
Body Mass Index	8%
Closest Estimated Glomerular Filtration Rate	0%
Urine Albumin Creatinine Ratio	37%
History of past/current smoking	25%
Low density lipoprotein cholesterol	30%
High density lipoprotein cholesterol	30%
Triglycerides	30%
Systolic Blood pressure	1%
Diastolic Blood pressure	1%
Serum Albumin	21%
Hemoglobin	23%
Platelet Count	24%
HbA_{1c}	20%
Liver enzymes (AST)	21%
Calcium	16%

ESM Table 3. Percent of patients in each strata of KDIGO risk from A) NHANES and B) KidneyIntelX study cohort

**A. Percent of patients in each strata from NHANES[1]** (Number and Percent of US Population with T2D and Intended Use for the KidneyIntelX test)							**Sub Totals**
**Albuminuria Categories**				A1 < 3mg/mmol	A2 3-29 mg/mmol	A3 ≥ 30 mg/mmol	
**GFR categories (ml/min)**	**G1**	Normal or high	≥ 90	N/A	20%	4%	24%
	**G2**	Mildly Decreased	60-89	N/A	18%	8%	26%
	G3a	Mildly to moderately decreased	45-59	25%	8%	5%	38%
	G3b	Moderately to severely decreased	30-44	5%	4%	3%	12%
**Sub-Totals**				30%	50%	20%	100%

**B. Percent of Patients in each Strata from KidneyIntelX Study Population**							**Sub Totals**
**Albuminuria Categories**				A1 < 3mg/mmol	A2 3-29 mg/mmol	A3 ≥ 30 mg/mmol	
**GFR categories (ml/min)**	**G1**	Normal or high	≥ 90	N/A	13%	3%	16%
	**G2**	Mildly Decreased	60-89	N/A	22%	8%	30%
	G3a	Mildly to moderately decreased	45-59	18%	10%	6%	34%
	G3b	Moderately to severely decreased	30-44	9%	6%	5%	20%
**Sub-Totals**				28%	51%	22%	100%

ESM Table 4. Test characteristics by KDIGO and KidneyIntelX Risk Categories

Training

With UACR present (N=417)

**Risk Category***	**KDIGO**			**Risk Category**	**KidneyIntelX**		
**Population**	**Sens**	**Spec**	**NPV**	**Population**	**Sens**	**Spec**	**NPV**
**Moderate CKD Risk (Yellow) (58%)**	55%	62%	83%	**Low risk (42%)**	89%	50%	94%
**Population**	**Sens**	**Spec**	**PPV**	**Population**	**Sens**	**Spec**	**PPV**
**Very High CKD Risk (Red) (15%)**	23%	87%	33%	**High risk (16%)**	47%	92%	63%

With UACR Imputed to 10 (N=686)

**Risk Category***	**KDIGO**			**Risk Category**	**KidneyIntelX**		
**Population**	**Sens**	**Spec**	**NPV**	**Population**	**Sens**	**Spec**	**NPV**
**Moderate CKD Risk (Yellow) (60%)**	49%	62%	83%	**Low risk (45%)**	88%	53%	94%
**Population**	**Sens**	**Spec**	**PPV**	**Population**	**Sens**	**Spec**	**PPV**
**Very High CKD Risk (Red) (9%)**	15%	92%	33%	**High risk (15%)**	46%	93%	61%

Validation

With UACR Present (N=296)

Risk Category*	KDIGO			Risk Category	KidneyIntelX		
**Population**	**Sens**	**Spec**	**NPV**	**Population**	**Sens**	**Spec**	**NPV**
**Moderate CKD Risk (Yellow) (53%)**	67%	59%	85%	**Low risk (45%)**	87%	54%	93%
**Population**	**Sens**	**Spec**	**PPV**	**Population**	**Sens**	**Spec**	**PPV**
**Very High CKD Risk (Red) (16%)**	28%	88%	40%	**High risk (17%)**	51%	93%	69%

With UACR Imputed to 10 (N=460)

Risk Category*	KDIGO			Risk Category	KidneyIntelX		
**Population**	**Sens**	**Spec**	**NPV**	**Population**	**Sens**	**Spec**	**NPV**
**Moderate Risk (Yellow) (59%)**	53%	63%	82%	**Low risk (46%)**	81%	54%	91%
**Population**	**Sens**	**Spec**	**PPV**	**Population**	**Sens**	**Spec**	**PPV**
**Very High Risk (Red) (10%)**	18%	92%	40%	**High risk (16%)**	45%	92%	62%

*see page 6 in KDIGO 2012 Clinical Practice Guideline for the Evaluation and Management of Chronic Kidney Disease; Kidney International Supplements 2013 for heat map describing strata “moderately increased risk”, “high risk” and “very high risk”

ESM Table 5. Net Reclassification from KDIGO Risk Strata to KidneyIntelX Risk Strata in Derivation and Validation

NRI from KDIGO Strata to KidneyIntelX Strata in Derivation

		KidneyIntelX Score			*Total*
	**KDIGO Strata**	***Low***	***Intermediate***	***High***	
**Composite Kidney Event**	**Moderately Increased Risk**	15	35	20	70
	**High Risk**	1	17	28	46
	**Very High Risk**	1	5	15	21
	**Total Events**	17	57	63	**137**
**No Kidney Event Count**	**Moderately Increased Risk**	198	126	19	343
	**High Risk**	79	73	12	164
	**Very High Risk**	13	20	9	42
	**Total Non-events**	290	219	40	**549**

Reclassified

	Higher n	Higher %	Lower n	Lower %	
**Event**	83	60.6	7	5.1	55.5
**Non-event**	157	28.6	112	20.4	-8.2

NRI from KDIGO Strata to KidneyIntelX Strata in Validation

		KidneyIntelX Score			*Total*
	**KDIGO Strata**	***Low***	***Intermediate***	***High***	
**Composite Kidney Event**	**Moderately Increased Risk**	14	21	14	49
	**High Risk**	6	12	18	36
	**Very High Risk**	0	4	15	19
	**Total Events**	20	37	47	**104**
**No Kidney Event Count**	**Moderately Increased Risk**	132	80	11	223
	**High Risk**	53	39	13	105
	**Very High Risk**	7	16	5	28
	**Total Non-events**	192	135	29	**356**

Reclassified

	Higher n	Higher %	Lower n	Lower %	
**Event**	53	51.0	10	9.6	41.3
**Non-event**	104	29.2	76	21.3	-7.9

*see page 6 in KDIGO 2012 Clinical Practice Guideline for the Evaluation and Management of Chronic Kidney Disease; Kidney International Supplements 2013 for heat map describing strata “moderately increased risk”, “high risk” and “very high risk”

ESM Fig. 1. Selection of Cohorts

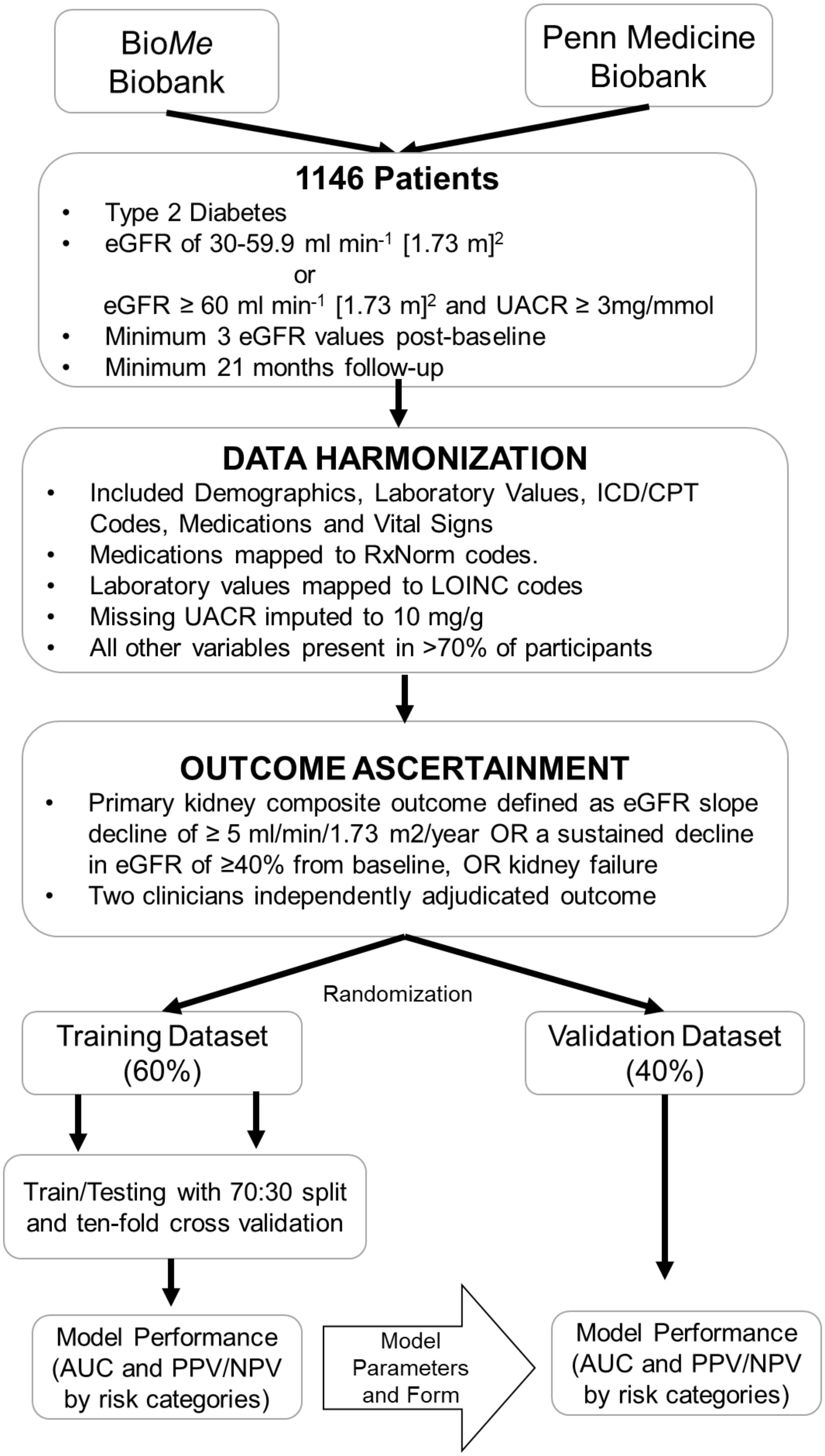

ESM Fig. 2. Observed vs. Expected (calibration plot) for the Composite Kidney Outcome in Derivation (Panel A) and Validation (Panel B)

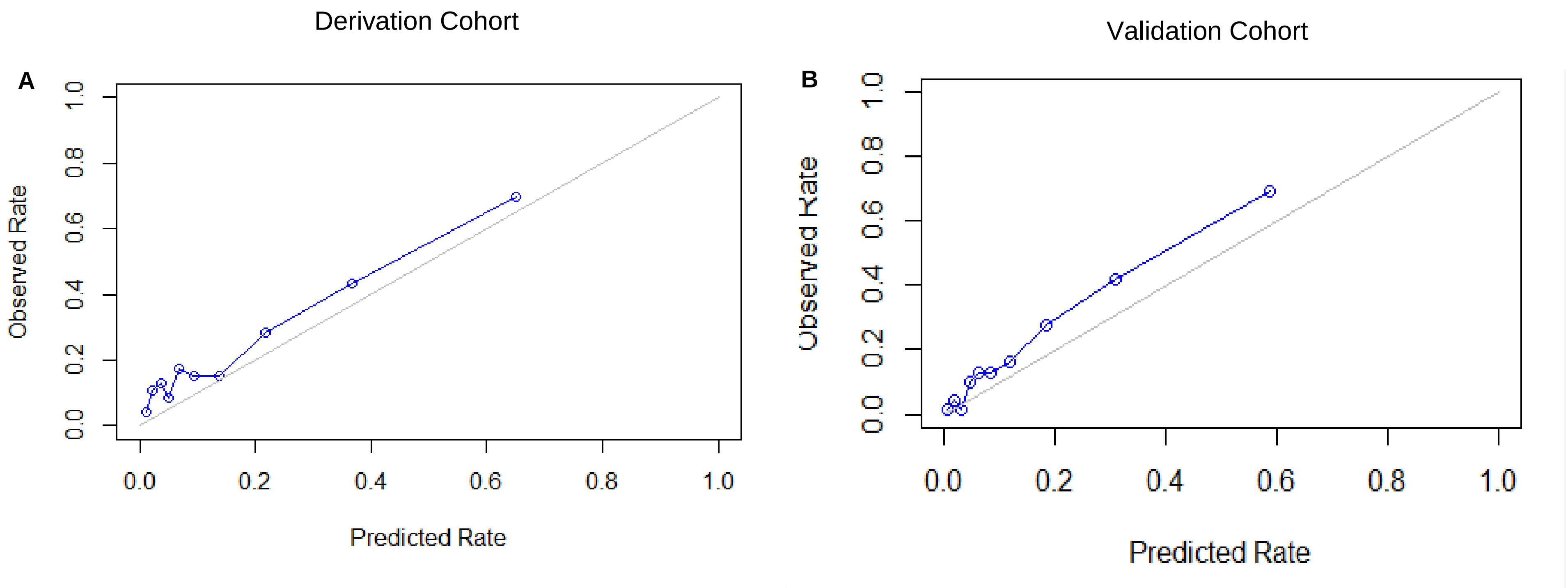

ESM Fig. 3. Kaplan-Meier Curves by KDIGO Risk Strata for the Endpoint of Sustained 40% Decline in eGFR or Kidney Failure in Derivation (Panel A) and Validation (Panel B)

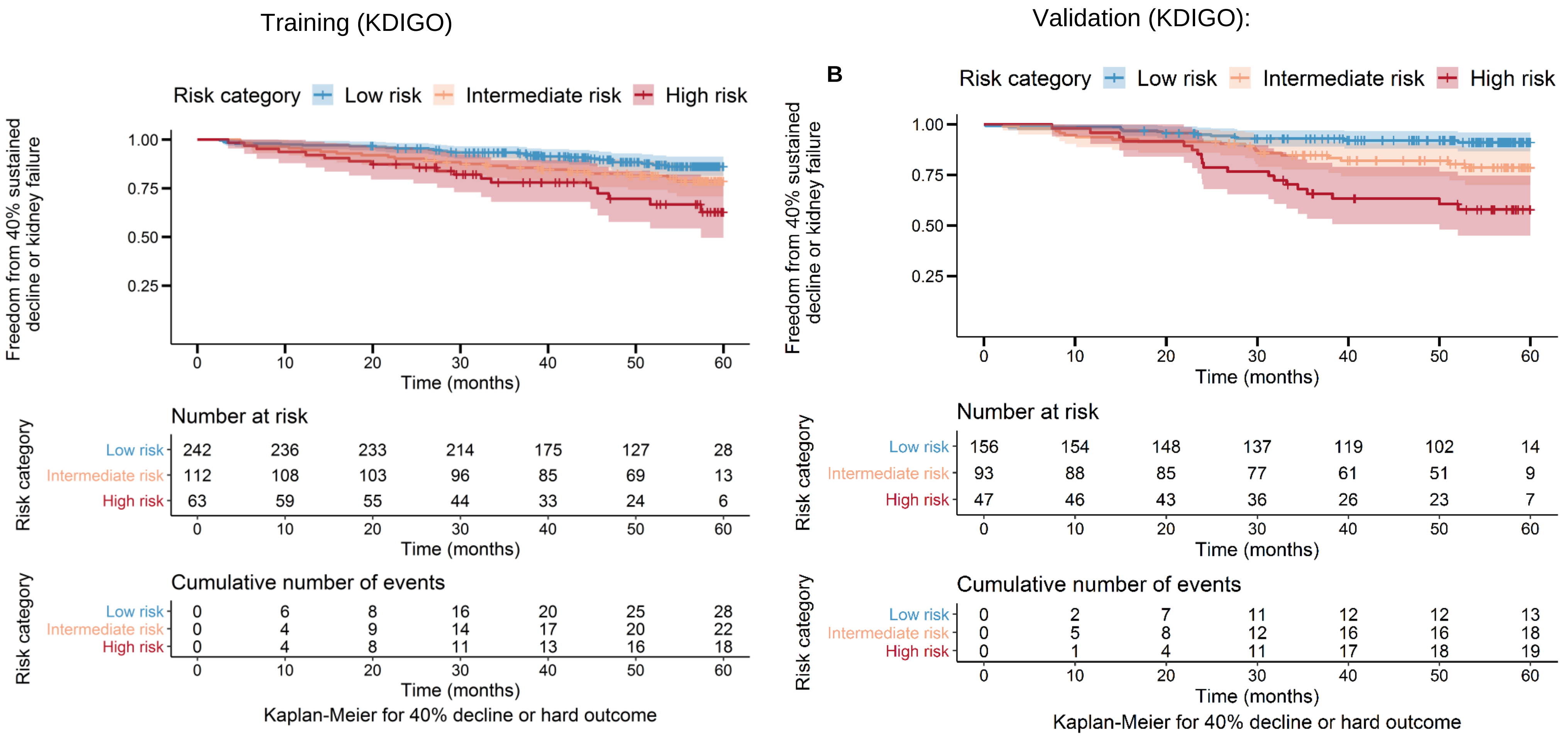

Low risk encompasses “moderate risk” KDIGO risk category, intermediate encompasses “high risk” CKD KDIGO risk category, and high risk encompasses “very high risk” KDIGO risk category

ESM Fig. 4. Kaplan-Meier Curves by KidneyIntelX Risk Strata for the Endpoint of Sustained 40% Decline in eGFR or Kidney Failure in Validation (Panel A) and in patients with data ≥ 5 years (Panel B)

KM plot for all patients in validation

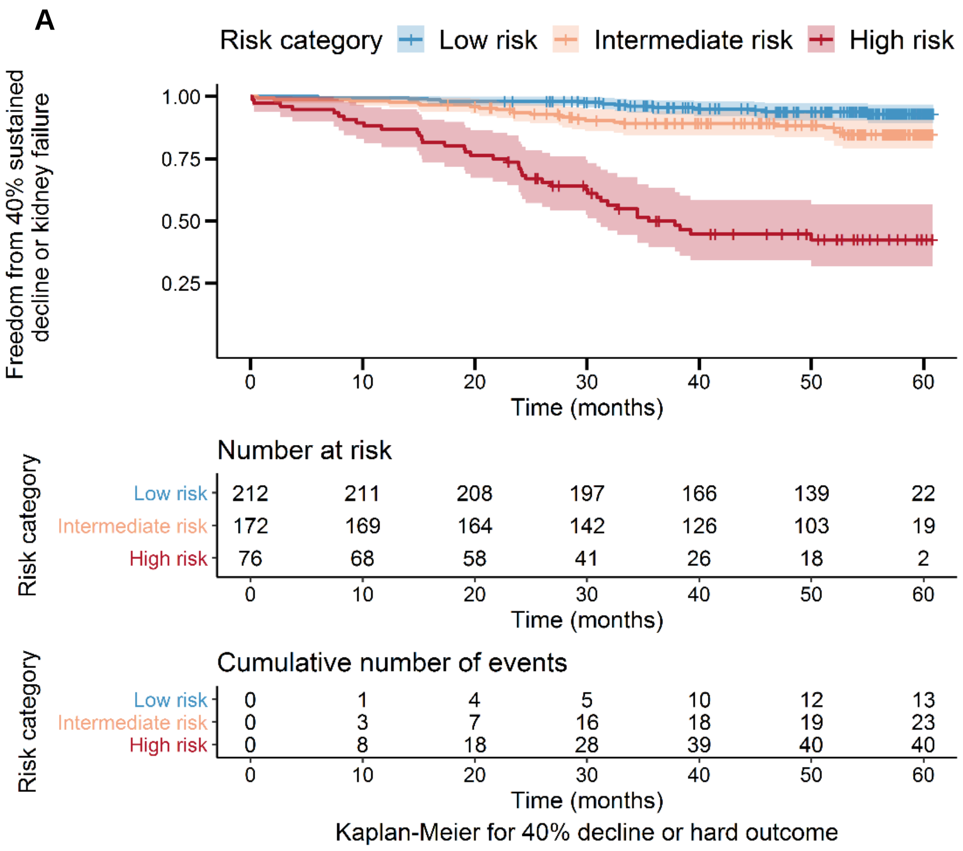

KM plot for patients with data ≥ 5 years (ensures alive for at least 5 years)

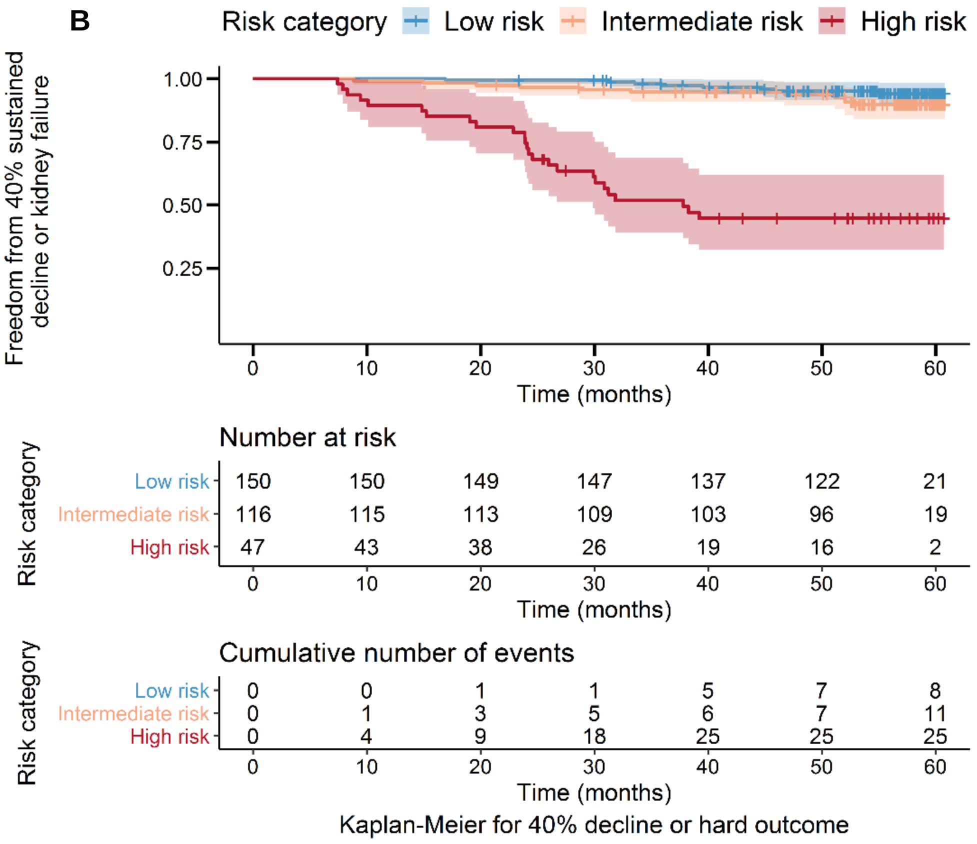

1. Centers for Disease Control and Prevention (CDC). National Center for Health Statistics (NCHS). National Health and Nutrition Examination Survey Data. Hyattsville, MD: U.S. Department of Health and Human Services, Centers for Disease Control and Prevention, 2018-2019. at https://www.cdc.gov/nchs/nhanes/index.htm.)
